# Subcutaneous Zygomycosis Successfully Treated With Itraconazole Monotherapy: A Case Report From Central India

**DOI:** 10.7759/cureus.113694

**Published:** 2026-07-30

**Authors:** Saurabh D Kose, Kirtee Meshram, Sushil Pande, Minal Jogi

**Affiliations:** 1 Department of Dermatology, N. K. P. Salve Institute of Medical Sciences and Research Centre and Lata Mangeshkar Hospital, Nagpur, IND

**Keywords:** basidiobolomycosis, basidiobolus ranarum, deep fungal infection, eosinophilic granuloma, itraconazole, splendore-hoeppli phenomenon, subcutaneous zygomycosis

## Abstract

Subcutaneous zygomycosis, also known as basidiobolomycosis, is a rare, chronic fungal infection that predominantly affects immunocompetent individuals in tropical and subtropical regions. The disease often presents as firm, painless, indurated subcutaneous swellings that may mimic malignancy, chronic bacterial infections, tuberculosis, or other deep fungal infections, leading to frequent misdiagnosis and unnecessary surgical interventions. We report the case of a 53-year-old immunocompetent man who presented with gradually enlarging, painless swellings over the right thigh and groin, accompanied by mild intermittent burning and pinprick pain for three months. There was no history of trauma or systemic illness. Examination revealed multiple firm, non-tender, indurated plaques with surface ulceration. Laboratory investigations were unremarkable. Histopathological examination of a wedge biopsy demonstrated dense eosinophilic granulomatous inflammation and broad, yeast-like fungal elements encased within an eosinophilic matrix (Splendore-Hoeppli phenomenon), confirming basidiobolomycosis. Fungal culture identified *Basidiobolus ranarum*. The patient was treated with suprabioavailable itraconazole at a dose of 65 mg twice daily. Marked clinical improvement was observed within one month, and complete resolution of the lesions occurred by five months, leaving minimal residual hyperpigmentation. No recurrence was noted during follow-up. This case highlights the importance of early biopsy and mycological evaluation in patients with chronic subcutaneous swellings to avoid misdiagnosis and unnecessary surgery. Itraconazole is an effective and well-tolerated therapy for subcutaneous zygomycosis, offering a nonsurgical treatment option. Early recognition and antifungal therapy can ensure excellent outcomes in this elusive infection.

## Introduction

Zygomycosis refers to a spectrum of fungal infections caused by organisms historically classified under the class Zygomycetes, which historically included two clinically significant orders: Mucorales and Entomophthorales [1]. Mucorales are typically associated with aggressive, invasive infections, such as mucormycosis, in immunocompromised individuals and are characterized by rapid tissue necrosis and high mortality rates [2]. In contrast, Entomophthorales, particularly *Basidiobolus ranarum*, cause chronic, localized subcutaneous disease in immunocompetent hosts [3]. While Mucorales (e.g., *Rhizopus* and *Mucor*) are the predominant cause of mucormycosis in India, Entomophthorales are responsible for subcutaneous zygomycosis, which is endemic in tropical and subtropical regions, including India, Africa, and Southeast Asia [4,5]. Subcutaneous zygomycosis caused by *Basidiobolus ranarum* is also referred to as subcutaneous basidiobolomycosis, and the two terms are commonly used interchangeably in the clinical literature. In India, subcutaneous basidiobolomycosis is increasingly recognized, especially in the southern states, but cases have been reported across the country [6,7]. The infection is often linked to minor trauma, insect bites, or the implantation of spores from contaminated soil or vegetation and is more common in children and young adults, with a male predominance [3,4].

The clinical diagnosis of subcutaneous basidiobolomycosis is challenging because of its indolent presentation. Patients typically develop chronic, painless, firm, indurated plaques or swellings that progress slowly over weeks to months. These tumor-like lesions can mimic a variety of conditions, including tuberculosis, soft-tissue sarcoma, panniculitis, actinomycosis, sporotrichosis, chromoblastomycosis, and eumycetoma [5,7]. Misdiagnosis is common, and patients may undergo unnecessary surgical interventions or prolonged empirical therapy for other infections before the correct diagnosis is established. The disease is often mistaken for malignancy or chronic bacterial infection, especially in endemic regions where tuberculosis is prevalent [8].

This case is clinically noteworthy because the patient presented with multiple indurated plaques at separate sites on the thigh and groin, including an ulcerated lesion that clinically resembled a chronic infectious or neoplastic process. The diagnosis was supported by characteristic histopathological findings and confirmed by fungal culture, which identified *Basidiobolus ranarum* [6]. The patient achieved complete resolution with oral suprabioavailable itraconazole alone, without the need for surgery [5]. This underscores the importance of early clinicopathological diagnosis and highlights the effectiveness of itraconazole as a noninvasive treatment option for subcutaneous basidiobolomycosis [7].

This case was previously presented as an e-poster at DERMACON Jaipur 2025 on February 7, 2025.

## Case presentation

**Figure 1:**
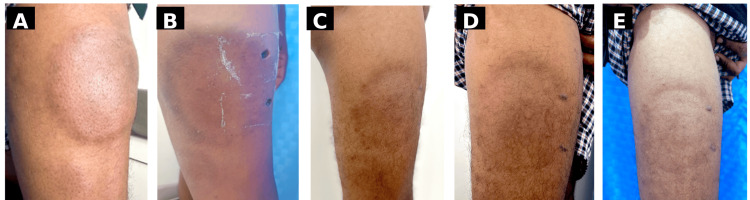
Serial clinical response of the lateral thigh lesion to itraconazole therapy. Serial clinical photographs showing the progressive response of subcutaneous zygomycosis involving the lateral aspect of the right thigh to oral itraconazole therapy. (A) Baseline image showing a firm, well-defined, indurated subcutaneous swelling over the lateral thigh. (B) First follow-up showing reduced swelling and induration with surface scaling. (C) Second follow-up showing further flattening of the lesion with residual post-inflammatory hyperpigmentation. (D) Third follow-up showing marked clinical improvement with minimal residual induration and biopsy-site changes. (E) Final follow-up showing complete clinical resolution with residual post-inflammatory hyperpigmentation and minor scarring.

**Figure 2:**
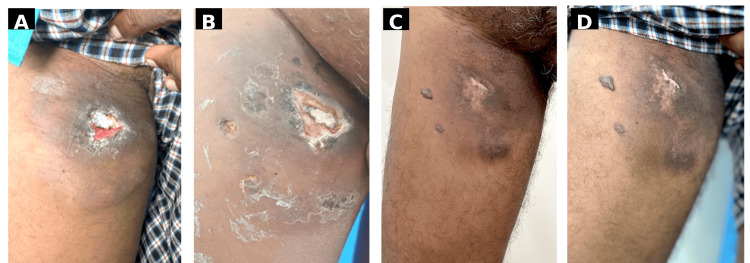
Serial clinical response of the right groin plaque to itraconazole therapy. Serial clinical photographs showing progressive improvement in subcutaneous zygomycosis involving the right groin in response to oral itraconazole therapy. (A) Baseline image showing an indurated groin plaque with superficial ulceration, crusting, and surrounding hyperpigmentation. (B) First follow-up showing persistent ulceration with surrounding scaling and reduced inflammatory induration. (C) Second follow-up showing healing of the ulceration with residual post-inflammatory hyperpigmentation and keloidal changes. (D) Final follow-up showing complete clinical resolution with residual post-inflammatory hyperpigmentation and scarring.

**Figure 3:**
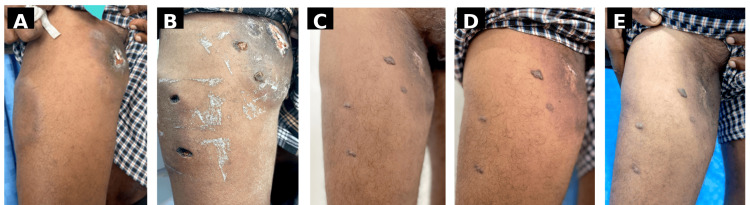
Serial clinical response of the thigh and groin lesions to itraconazole therapy. Serial clinical photographs showing progressive improvement in subcutaneous zygomycosis involving the thigh and groin in response to oral itraconazole therapy. (A) Baseline image showing firm, indurated plaques over the thigh and groin, with superficial ulceration and surrounding hyperpigmentation. (B) First follow-up showing persistent crusted lesions with surrounding scaling and reduced induration. (C) Second follow-up showing healing of the ulcerated areas with residual post-inflammatory hyperpigmentation and biopsy-site changes. (D) Third follow-up showing further flattening of the lesions with residual pigmentation and scarring. (E) Final follow-up showing complete clinical resolution with residual post-inflammatory hyperpigmentation and minor scarring.

A 53-year-old immunocompetent man, a tea vendor by occupation with well-controlled hypertension, presented with a three-month history of gradually enlarging, painless swellings over the right posterolateral thigh (Figure 1), groin (Figure 2), and lateral thigh (Figure 3). He denied regular occupational exposure to soil, decaying vegetation, reptiles, amphibians, or livestock. He also did not recall any preceding trauma, insect bite, or implantation injury. The lesions began as pea-sized nodules and progressively enlarged into firm, indurated plaques. The patient reported mild intermittent burning and pinprick pain but denied any history of trauma, other systemic illness, or immunosuppressive conditions. He had no fever or other constitutional symptoms.

On examination, three well-defined, firm, non-tender, indurated plaques were noted, ranging in size from 3 × 4 cm to 6 × 8 cm, with the largest lesion over the right groin showing superficial ulceration. The overlying skin was hyperpigmented in places. No regional lymphadenopathy was detected. Systemic examination was unremarkable, and there were no signs of disseminated disease.

Given the chronic, indurated nature of the swellings and the presence of ulceration, the clinical differential diagnoses included soft-tissue tumors, such as liposarcoma, cutaneous tuberculosis, actinomycosis, sporotrichosis, chromoblastomycosis, panniculitis, and other deep fungal infections. The tumor-like presentation and chronicity raised suspicion for both neoplastic and infectious etiologies, as is often reported in similar cases.

Routine laboratory investigations, including a complete blood count, liver and renal function tests, and blood glucose levels, were within normal limits. Serologic testing for HIV and HCV and testing for hepatitis B surface antigen (HBsAg) were negative. A wedge biopsy was obtained from a lesion.

**Figure 4:**
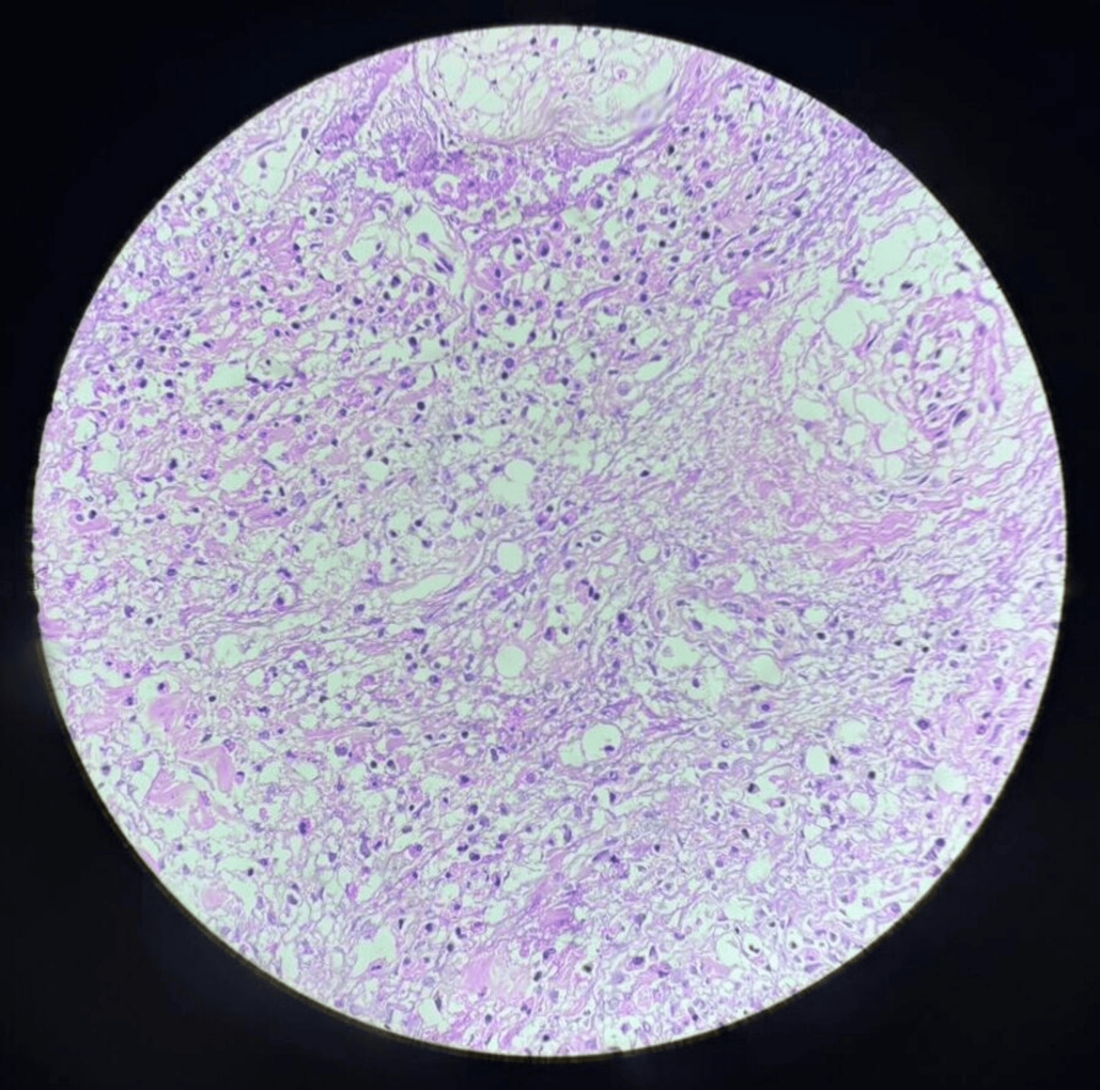
Histopathological findings in subcutaneous zygomycosis. Photomicrograph of a skin biopsy showing eosinophil-rich granulomatous inflammation involving the subcutaneous tissue, with histiocytes and multinucleated giant cells (H&E staining, ×100 magnification).

**Figure 5:**
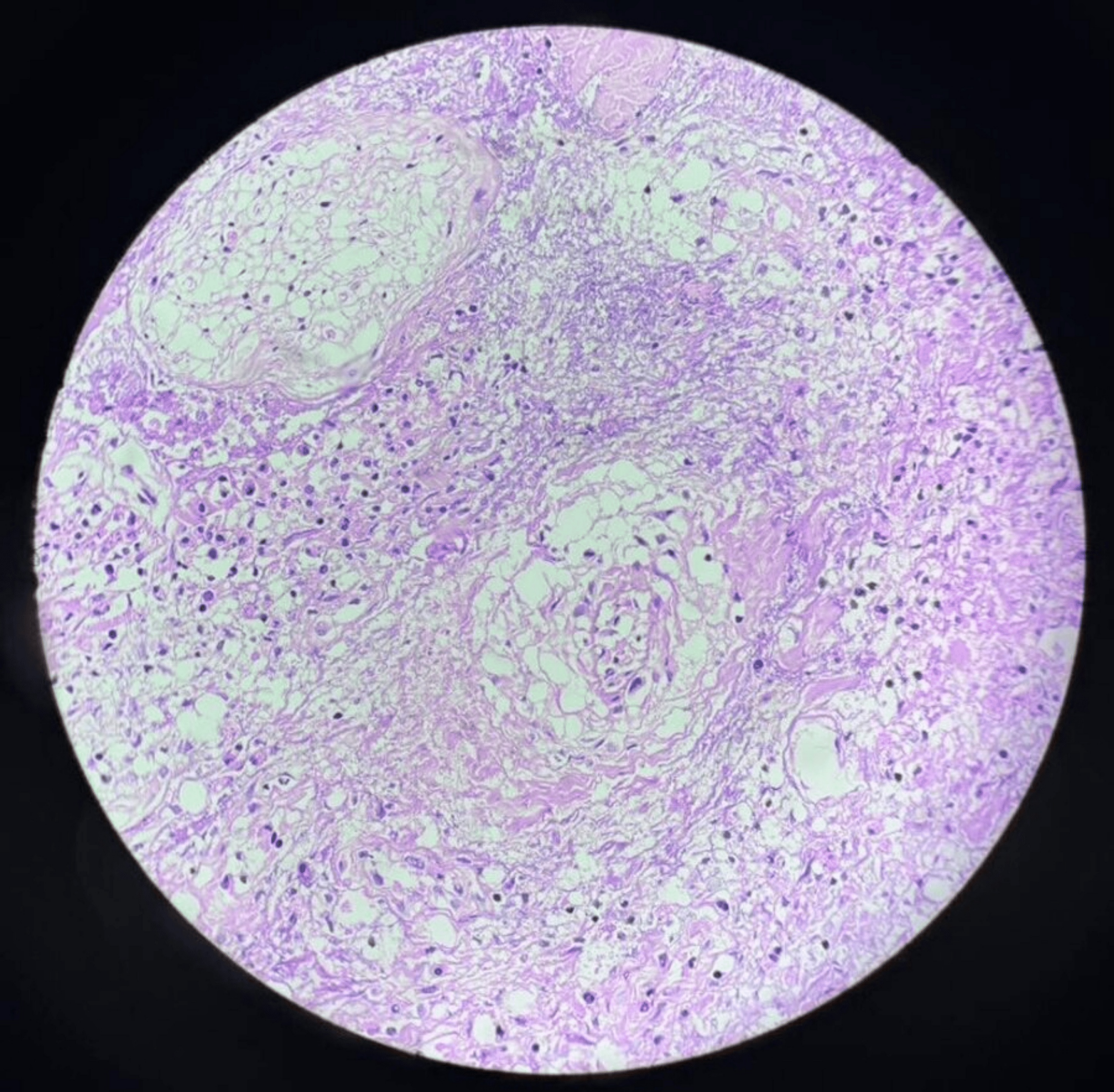
Histopathological findings on H&E staining. Histopathological examination showing eosinophil-rich granulomatous inflammation in the subcutaneous tissue, with multinucleated giant cells and broad fungal hyphae surrounded by eosinophilic material, consistent with the Splendore-Hoeppli phenomenon (H&E staining, ×100 magnification).

**Figure 6:**
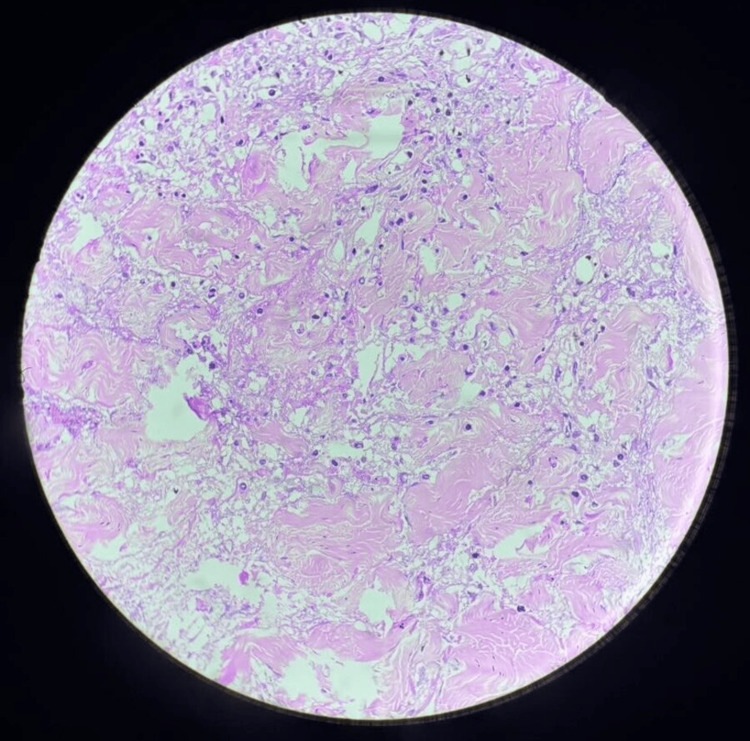
Histopathological findings on H&E staining. Histopathological examination showing dense fibrocollagenous tissue with chronic granulomatous inflammation and an eosinophilic infiltrate, consistent with subcutaneous basidiobolomycosis (H&E staining, ×100 magnification).

Histopathological examination of the wedge biopsy (Figure 4) showed hyperkeratotic squamous epithelium with focal parakeratosis and dense eosinophil-rich granulomatous inflammation. Broad fungal elements surrounded by eosinophilic material were observed, consistent with the Splendore-Hoeppli phenomenon (Figures 5-6). These findings were strongly suggestive of subcutaneous basidiobolomycosis. Special stains, including periodic acid-Schiff (PAS) and Gomori methenamine silver (GMS), highlighted the presence of broad, thin-walled, aseptate fungal hyphae within the granulomatous tissue, further supporting the diagnosis. Fungal culture identified *Basidiobolus ranarum*.

Baseline liver function tests (LFTs) were performed and were within normal limits. The patient was started on suprabioavailable itraconazole at a dose of 65 mg twice daily, administered after meals in accordance with standard recommendations to enhance absorption. Suprabioavailable itraconazole was selected because it provides more predictable absorption and has reduced dependence on gastric acidity compared with conventional itraconazole capsules. Treatment was initiated at presentation, after three months of progressive disease, and continued for five months. The patient was monitored monthly through clinical reviews and repeat LFTs to assess for potential hepatotoxicity associated with azole antifungal therapy. Marked regression was observed after one month of therapy, and complete clinical resolution was achieved at the end of five months. The patient tolerated the formulation well, and no adverse effects were reported during treatment. After completing treatment, the patient was followed for an additional six months, with no evidence of recurrence. Importantly, surgical intervention was avoided because of the excellent clinical response to medical therapy alone.

At the one-month follow-up, the lesions showed marked regression, with reductions in induration and symptoms. By five months, the lesions had completely resolved, leaving mild residual hyperpigmentation and minor keloidal changes at the biopsy site. No recurrence was noted during the six-month follow-up period after completion of therapy.

## Discussion

This case highlights the diagnostic challenge of subcutaneous zygomycosis in immunocompetent individuals, especially in tropical regions, where chronic indurated swellings are often clinically attributed to tuberculosis, soft-tissue tumors, or other deep fungal infections [4]. The indolent, tumor-like presentation of subcutaneous basidiobolomycosis frequently leads to misdiagnosis and unnecessary surgical interventions, as reported in multiple cases from India and other endemic regions [7,9].

Subcutaneous zygomycosis is caused by *Basidiobolus ranarum*, a saprophytic fungus belonging to the order Entomophthorales. The organism is widely distributed in soil, decaying organic matter, and the GI tracts of amphibians and reptiles [3]. The infection is endemic in tropical and subtropical regions, including India, Africa, and Southeast Asia, and predominantly affects immunocompetent children and young adults, although individuals of any age may be affected [7,9]. The presumed mode of transmission is the traumatic implantation of spores following minor trauma, insect bites, or contact with contaminated vegetation. Common sites of involvement include the thighs, buttocks, trunk, and extremities, reflecting areas prone to minor injuries [3,7-10].

Clinically, subcutaneous basidiobolomycosis presents as a chronic, painless, firm subcutaneous swelling or indurated plaque that progresses slowly over weeks to months [5]. The lesions are typically non-tender, with the overlying skin often appearing hyperpigmented or showing ulceration in advanced cases [6]. Systemic symptoms are usually absent, and regional lymphadenopathy is uncommon [1]. The slow progression and absence of systemic features often result in the condition being mistaken for neoplastic or chronic infectious processes, such as soft-tissue sarcoma, cutaneous tuberculosis, actinomycosis, sporotrichosis, chromoblastomycosis, eumycetoma, or panniculitis [7].

Pathologically, the hallmark of subcutaneous basidiobolomycosis is eosinophil-rich granulomatous inflammation with broad, thin-walled, infrequently septate fungal elements [4]. The Splendore-Hoeppli phenomenon, characterized by eosinophilic material surrounding the fungal hyphae, is a characteristic finding that aids in diagnosis [1]. Special stains, such as PAS and GMS, highlight the fungal elements, while culture on Sabouraud dextrose agar confirms the diagnosis by demonstrating the typical creamy, radially folded colonies of *Basidiobolus ranarum* [4]. However, cultures may not always be positive, making histopathology crucial for diagnosis in resource-limited settings.

The main clinical differential diagnoses include soft-tissue sarcoma, cutaneous tuberculosis, actinomycosis, sporotrichosis, chromoblastomycosis, eumycetoma, panniculitis, and other chronic granulomatous disorders. The tumor-like appearance and chronicity often lead to unnecessary surgical excisions or prolonged empirical therapy for tuberculosis or bacterial infections before the correct diagnosis is established [4].

Historically, potassium iodide was considered the gold standard for treating subcutaneous zygomycosis, with numerous reports documenting dramatic responses [8]. However, azole antifungals, particularly itraconazole, have emerged as preferred treatment options because of their tissue penetration, tolerability, and efficacy [4]. The duration of therapy is individualized based on the clinical response, but most cases require several months of treatment [8,10]. Surgical excision alone is generally unnecessary and may be disfiguring or may even promote the spread of infection [5,8]. In our case, the patient responded excellently to suprabioavailable itraconazole at a dose of 65 mg twice daily, with marked regression at one month and complete resolution by five months, thereby avoiding the need for surgery.

The favorable response in our patient emphasizes that timely biopsy, recognition of characteristic histopathological findings, and early antifungal treatment can lead to complete resolution and prevent unnecessary surgical interventions. Increased awareness among clinicians, especially in endemic regions, is essential to avoid misdiagnosis and ensure optimal outcomes for this rare but treatable infection.

## Conclusions

Subcutaneous zygomycosis should be considered in patients presenting with chronic, firm, indurated subcutaneous plaques or swellings, particularly when the lesions progress slowly and fail to respond to routine antibacterial or anti-inflammatory therapy. Its tumor-like clinical appearance may create diagnostic confusion with soft-tissue neoplasms, cutaneous tuberculosis, panniculitis, and other deep fungal infections. Early clinical suspicion is therefore essential to avoid diagnostic delays and unnecessary invasive procedures.

This case highlights the value of timely tissue biopsy, histopathological examination, special fungal stains, and fungal culture in confirming the diagnosis. Recognition of eosinophil-rich granulomatous inflammation with broad fungal elements and the Splendore-Hoeppli phenomenon provided an important diagnostic clue. Identification of *Basidiobolus ranarum* on fungal culture further supported the diagnosis in our patient.

Prompt initiation of itraconazole led to progressive regression of the lesions and complete clinical resolution without the need for surgical intervention. This case reinforces that early recognition and appropriate antifungal therapy can result in favorable outcomes while minimizing morbidity and avoiding disfiguring surgery.

Further studies are needed to define the optimal duration of therapy, the role of suprabioavailable itraconazole formulations, and the clinical predictors of treatment response and recurrence.
